# Isoflurane, like sepsis, decreases CYP1A2 liver enzyme activity in intensive care patients: a clinical study and network model

**DOI:** 10.1186/s40635-024-00617-8

**Published:** 2024-04-08

**Authors:** Thomas Köhler, Elke Schwier, Janina Praxenthaler, Carmen Kirchner, Günther Winde, Björn Koos, Dietrich Henzler

**Affiliations:** 1 Department of Anesthesiology, Surgical Intensive Care, Emergency and Pain Medicine, Ruhr University Bochum, Klinikum Herford, Herford, Germany; 2 Department of Anesthesiology, Intensive Care and Pain Medicine, Southeast Bavaria Hospitals, Klinikum Traunstein, Traunstein, Germany; 3 Department of General and Visceral Surgery, Thoracic Surgery and Proctology, Ruhr University Bochum, Klinikum Herford, Herford, Germany; 4 Department of Anesthesiology, Intensive Care and Pain Medicine, Ruhr University Bochum, Knappschaftskrankenhaus Bochum GmbH, Bochum, Germany; 5 Department of Anesthesiology and Intensive Care Medicine, AMEOS-Klinikum Halberstadt, Academic Teaching Hospital, Gleimstraße 5, 38820 Halberstadt, Germany

**Keywords:** Isoflurane, Sepsis, Cytochrome P450 1A2, LiMAx, Maximum liver function capacity test, Hypoxia inducible factor

## Abstract

**Purpose:**

Liver function of intensive care patients is routinely monitored by static blood pathology. For specific indications, liver specific cytochrome activity may be measured by the commercially available maximum liver function capacity (LiMAx) test via quantification of the cytochrome P450 1A2 (CYP1A2) dependent C-methacetin metabolism. Sedation with the volatile anesthetic isoflurane was suspected to abrogate the correlation of LiMAx test with global liver function. We hypothesized that isoflurane has a CYP1A2-activity and LiMAx test result decreasing effect.

**Methods:**

In this monocentric, observational clinical study previously liver healthy intensive care patients, scheduled to be changed from propofol to isoflurane sedation, were enrolled. LiMAx testing was done before, during and after termination of isoflurane sedation.

**Results:**

The mean LiMAx value decreased during isoflurane sedation. Septic patients (*n* = 11) exhibited lower LiMAx values compared to non-septic patients (*n* = 11) at all time points. LiMAx values decreased with isoflurane from 140 ± 82 to 30 ± 34 µg kg^−1^ h^−1^ in the septic group and from 253 ± 92 to 147 ± 131 µg kg^−1^ h^−1^ in the non-septic group while laboratory markers did not imply significant hepatic impairment. Lactate increased during isoflurane inhalation without clinical consequence.

**Conclusion:**

Sepsis and isoflurane have independently demonstrated an effect on reducing the hepatic CYP1A2-activity. A network model was constructed that could explain the mechanism through the influence of isoflurane on hypoxia inducible factor (HIF-1α) by upregulation of the hypoxia-inducible pathway and the downregulation of CYP1A2-activity via the ligand-inducible pathway. Thus, the increased anaerobic metabolism may result in lactate accumulation. The influence of isoflurane sedation on the validated correlation of global liver function with CYP1A2-activity measured by LiMAx testing needs to be investigated in more detail.

**Supplementary Information:**

The online version contains supplementary material available at 10.1186/s40635-024-00617-8.

## Introduction

Patients treated in the intensive care unit (ICU) are at high risk for liver injury and failure [1]. Static liver parameters provide information about direct, morphological liver cell damage. To gain information about the dynamic liver function, the activity of the liver specific, microsomal cytochrome P450 1A2 (CYP1A2) can be measured noninvasively and in real time by a commercially available test, the maximum liver function capacity (LiMAx) method [2]. By principle, labeled ^13^C-methacetin is metabolized in liver cells to paracetamol and ^13^CO_2_ which is quantified by exhaled breath analysis. The reaction used has been established to determine the CYP1A2-mediated metabolism in vitro [3].

Clinically, LiMAx values between 140 to 314 µg kg^−1^ h^−1^ correlate with limited hepatic impairment and may be the basis for dose adjustments of primary liver toxic drugs [4]. A LiMAx value below 140 µg kg^−1^ h^−1^ indicates significant hepatic injury while values < 100 µg kg^−1^ h^−1^ are associated with increased mortality in septic patients [5, 6]. Increasing LiMAx values may indicate recovery of liver function early, before normalization of static laboratory parameters and decreasing values may serve as a prognostic tool to facilitate the decision for urgent liver transplantation [7].

Previous observations indicated that simultaneous sedation with the volatile anesthetic isoflurane could cause a profound decrease in LiMAx test results that might not correlate with severe liver function impairment [8]. Isoflurane has been used in increasing numbers of patients since the introduction of the anesthetic conserving device (AnaConDa) that allows for safe application [9, 10]. While organ impairment in inhaled and propofol-sedated patients is similar, inhaled sedation with isoflurane has the advantage of shorter wake-up times, lower opioid consumption and faster establishment of spontaneous breathing [11]. It is also a viable second-line alternative if propofol should be terminated in long-term sedation for lipid overloading or prevention of mitochondrial damage.

A better knowledge about the potential influence of isoflurane on LiMAx testing would be essential in avoiding false decisions. We hypothesized that isoflurane sedation in mechanically ventilated ICU patients decreases CYP1A2-activity and concomitant LiMAx test results and lead to lactate increase. If so, we propose a conceivable underlying network model regarding liver cell metabolomics and biotransformation capacity that includes lactate accumulation.

## Methods

### Study design

The monocentric observational study was conducted in the mixed surgical ICU in a university hospital in Germany. The study was approved by the institutional ethics committee of the Ruhr-University Bochum (file number: 2020-661, dated 28 Aug 2020). It was registered on September 17th, 2020 in the German clinical trials register (DRKS-ID: DRKS00022228, https://drks.de/search/de/trial/DRKS00022228). The study has a both retrospective and prospective observational design. The article is written according to the STROBE guidelines [12].

### Patient population

Mechanically ventilated patients were eligible, if they were continuously sedated with intravenous propofol and were ordered to be switched to inhaled sedation with isoflurane by the treating physician for medical reasons. A change in sedation regimen was indicated, for example, when the maximum approved duration of therapy (7d) or the maximum dose for propofol was reached. Patients with sepsis or septic shock (septic patients) according to SEPSIS-3 criteria [13] were analyzed separately for likely influence on LiMAx testing. Patients with significant preexisting liver disease were excluded.

### Study procedure

We evaluated LiMAx test data retrospectively and prospectively from ICU patients of Apr 2019 to Dec 2020, when the necessary number of patients was reached. We planned to include 20 patients to gain significant results [14]. Thus 15 retrospective and 7 prospective patients were included into the study (Additional file 1: Fig. S1). The substitute decision-maker was informed about the study by an investigator, and written informed consent was obtained. If there was insufficient time between the physician decision to change sedation and the first LiMAx measurement to obtain written consent, enrollment in the study could be done after approval by an independent consulting physician.

In order to establish comparability of the LiMAx test values the retrospective data were matched to four time points also used for the prospective measurements: T0: before isoflurane sedation; T1, T2: measured on separate days during isoflurane sedation as part of routine clinical care; T3: after termination of isoflurane. The oxygen concentration used for mechanical ventilation was adjusted on the basis of arterial blood gas analyses and subsequently kept constant during LiMAx measurements (1 h).

Patient outcomes and characteristics including age, sex, height, weight, body mass index (BMI), medical history and diagnoses as well as laboratory parameters were obtained from the hospital information system. The physiological data were continuously documented using a patient data management system (Integrated Care Manager, Dräger, Lübeck, Germany).

For LiMAx testing, a Flip® Analyzer (Humedics GmbH, Berlin, Germany) was connected to the expiratory hose of the breathing circuit and measurements were performed as described previously [15]. In brief, 2 mg/kg body weight ^13^C-labeled methacetin (Humedics GmbH, Berlin, Germany) was administered intravenously. The ^12^C/^13^C ratio of the exhaled ^13^CO_2_ was quantified as a function of time as delta over baseline (DOB).

### Definition of outcomes

The primary outcome parameter was the microsomal hepatic function of CYP1A2 enzyme complex, measured by LiMAx testing. As secondary outcome, the serum lactate concentration (mmol/h) was determined at the four time points T0 to T3.

### Statistical analysis

Baseline data (T0) were compared between septic and non-septic patients using t-test or U-test, whichever was appropriate after testing for normality by using the KS-test (Table 1).

**Table 1:** Baseline characteristics

Parameter	All patients (*n* = 22)	With sepsis (*n* = 11)	Non-septic (*n* = 11)	*p*-value sepsis vs non-septic
Age (yr)	58 [49, 71]	56 [30, 73]	59 [51, 80]	0.3
Sex (*n*)				
Female	8	5	6	
Male	14	6	5	
Weight (kg)	90 [75, 99]	90 [60, 105]	90 [75, 95]	0.47
Height (cm)	179 [169, 180]	173 [166,180]	180 [178, 185]	0.29
BMI (kg/m^2^)	28 [25, 31]	30 [22, 31]	27 [26, 29]	0.68
SOFA score	10 [9, 12]	11 [10, 13]	9 [8, 10]	0.01
APACHE II score	22 [19, 26]	26 [18, 33]	21 [19, 24]	0.15
28-day mortality (*n*)	3 (14%)	1 (9%)	2 (18%)	0.56
Duration of propofol sedation before isoflurane (h)	51 [20, 87]	51 [46, 72]	48 [20, 88]	0.53
Duration of isoflurane sedation (h)	151 [71, 238]	164 [128, 243]	85 [50, 234]	0.17
Time interval T0–T3 (h)	263 [167, 459]	336 [179, 515]	242 [163, 379]	0.17
Time interval T0–T1 (h)	42 [24, 69]	56 [26, 97]	30 [24, 46]	0.116
Time interval T1–T2 (h)	43 [24, 96]	74 [25, 127]	25 [24, 70]	0.156
Time interval T2–T3 (h)	158 [104, 292]	147 [105, 359]	189 [95, 243]	0.758
Patients on drugs with CYP1A2				n/a
Inhibition (n)	7	3	4	
Induction (without PPI’s)(n)	10	6	4	
Admission diagnosis				n/a
Trauma	4	1	3	
Gastrointestinal disease	7	5	3	
Pulmonary disease	7	5	1	
Neurologic disorder	4	0	4	
Comorbidities (*n*)				n/a
Cardiovascular	12	7	5	
Pulmonary	6	5	1	
Diabetes	6	3	3	
Neurological	4	2	2	
BMI ≥ 35	1	0	1	

Parameters are reported as median [IQR]. Medication given at any time point: CYP1A2 inhibitors: fluoroquinolones. CYP1A2 inductors: insulin. All patients had received PPI’s (proton pump inhibitors). SOFA and APACHE II score were evaluated at T0. BMI: body mass index, SOFA: Sepsis-Related Organ Failure Assessment, APACHE II: Acute Physiology and Chronic Health Evaluation, CYP1A2: cytochrome P450 1A2

For the comparison of different timepoints, a linear mixed model [16] considering the influence of individual patients as a random effect was used (Table 2). For variables with significant time effects, multiple comparisons according to Tukey [17] were performed.

**Table 2:** Physiologic and pathologic data of all patients at the four study time points

	T0	T1	T2	T3	*p*-value
Isoflurane insp (%)	0 [0. 0]^*T1. T2^	0.33 [0.19, 0.39] ^*T0. T3^	0.35 [0.21, 0.55]^*T0. T3^	0 [0. 0]^*T1. T2^	< 0.001^*^
Isoflurane exp (%)	0 [0. 0]^*T1. T2^	0.65 [0.46, 0.81]^*T0, T3^	0.78 [0.55, 1.00]^*T0. T3^	0 [0. 0]^*T1. T2^	< 0.001^*^
LiMAx (µg kg^−1^ h^−1^)	177 [129, 298]^*T1. T2^	67 [15, 122]^*T0. T3^	49 [8, 165]^*T0.T3^	247 [152, 403]^*T1. T2^	< 0.001^*^
Bilirubin (mg/dl)	0.71 [0.28, 0.86]	0.46 [0.27, 0.79]	0.56 [0.30, 1.13]	0.67 [0.20, 1.22]	0.529
Creatinine (mg/dl)	0.77 [0.59, 1.26]	0.86 [0.54, 1.25]	0.79 [0.67, 0.97]	0.85 [0.63, 1.24]	0.345
INR	1.15 [1.04, 1.31]	1.14 [1.05, 1.30]	1.10 [1.03, 1.17]	1.09 [1.03, 1.14]	0.155
MELD score	9.7 [7.1, 22.8]	9.7 [7.2, 22.9]	8.4 [7.5, 21.9]	9.1 [7.3, 11.5]	0.067
AST (U/l)	36.5 [23.3, 90.3]	36.0 [26.5, 92.0]	33.0 [25.0, 58.0]	65.0 [44.3,103.5]	0.344
ALT (U/l)	29.0 [14.0, 78.0]	23.0 [13.5, 53.5]	19.0 [13.0, 30.0]	37.5 [25.3, 74.3]	0.288
AST/ALT ratio	1.14 [1.00, 1.89]	1.57 [0.92, 2.21]	1.56 [0.94, 2.21]	1.53 [1.05, 2.21]	0.313
Lactate (mmol/l)	1.13 [0.75, 1.31]	1.30 [0.86, 1.90]	1.39 [0.97, 1.78]	1.02 [0.87, 1.33]	0.123
Leucocytes (g/l)	9.9 [6.1, 18.3]	9.9 [8.5, 17.8]	12.5 [9.0, 14.3]	13.0 [9.0, 25.6]	0.519
Hemoglobin (g/l)	94 [90, 106]	89 [82, 102]	94 [82, 102]	87 [73, 95]	0.135
Hematocrit (%)	0.30 [0.27, 0.33]	0.28 [0.25, 0.31]	0.29 [0.27, 0.31]	0.27 [0.23, 0.30]	0.065
Thrombocytes (g/l)	216 [132, 271]	216 [97, 270]	225 [186, 303]	250 [217, 403]	0.084
MAP (mmHG)	77 [65, 85]	74 [71, 83]	77 [69, 91]	85 [69, 98]	0.425
Horowitz index	244 [173, 359]	283 [184, 336]	253 [189, 315]	240 [175, 333]	0.718
Noradrenalin (µg kg^−1^ min^−1^)	0.096 [0.025, 0.130]	0.107 [0.055, 0.255]^*T3^	0.100 [0.037, 0.194]^*T3^	0.000 [0.000, 0.047] ^*T1, *T2^	0.022*
SOFA score	10.0 [8.8, 12.0]^*T3^	10.5 [9.0, 12.3]^*T3^	10.0 [9.0, 11.0]^*T3^	8.0 [6.3, 10.0]^*T0, T1, T2^	0.001*

Parameters are reported as median [IQR]; *denotes significant difference vs. the indicated time point. *p*-value, *:significant. LiMAx: maximum liver function capacity; INR: international normalized ratio; MELD: model of end stage liver disease; AST: aspartate aminotransferase; ALT: alanine amino transferase; MAP: mean arterial pressure; SOFA: Sepsis-Related Organ Failure Assessment

The LiMAx and lactate measurements were analyzed as a function of isoflurane and the presence of sepsis or septic shock. The data collected were regarded as a repeated measures design [17]. Calculation of model parameters using restricted maximum likelihood was performed in GraphPad Prism version 9.3.1 (GraphPad Software, Boston, USA). The F-test was used to evaluate statistical significance of the influence variables with correction by Geisser–Greenhouse [17]. To ensure normal distribution and variance homogeneity of the residuals, the LiMax values were transformed using a root function, whereas lactate values were transformed using a natural logarithm.

## Results

Patient baseline characteristics are summarized in Table 1. All patients were mechanically ventilated. All patients received a proton pump inhibitor (PPI) that may be CYP1A2-inducting. The patients received neither amiodarone, diclofenac, (inhibitors) nor rifampicin, carbamazepine (inductors) (Table 1). Eleven patients had sepsis while the other eleven had non-septic pathologies. For analysis, patient data were divided into a septic and a non-septic group. The groups did not differ significantly in patient characteristics (Table 1) except that at T0, the septic patients showed more severe symptoms characterized by a higher Sepsis-Related Organ Failure Assessment (SOFA) score of 11 [13, 10] while non-septic patients had a SOFA score of 9 [8, 10] (*p* = 0.01). Five septic patients received continuous renal replacement therapy treatment.

The mean LiMAx start value for all patients decreased during isoflurane inhalation at both time points and increased back to pre-isoflurane sedation values after cessation of the inhalative agent (Table 2). There were no significant differences in LiMAx values between T0 and T3 (*p* = 0.201) or T1 and T2 (*p* = 0.995). No LiMAx measurement was available at T0 for 1 septic and 1 non-septic patient and at T2 for 2 septic patients (1 discharged, 1 missing measurement) and 2 non-septic patients (1 died, 1 discharged). At T3, 3 more patients had left the study (1 septic patient died, 2 non-septic patients discharged). Both patients that had died before T3 had received isoflurane until the end.

**Fig. 1:**
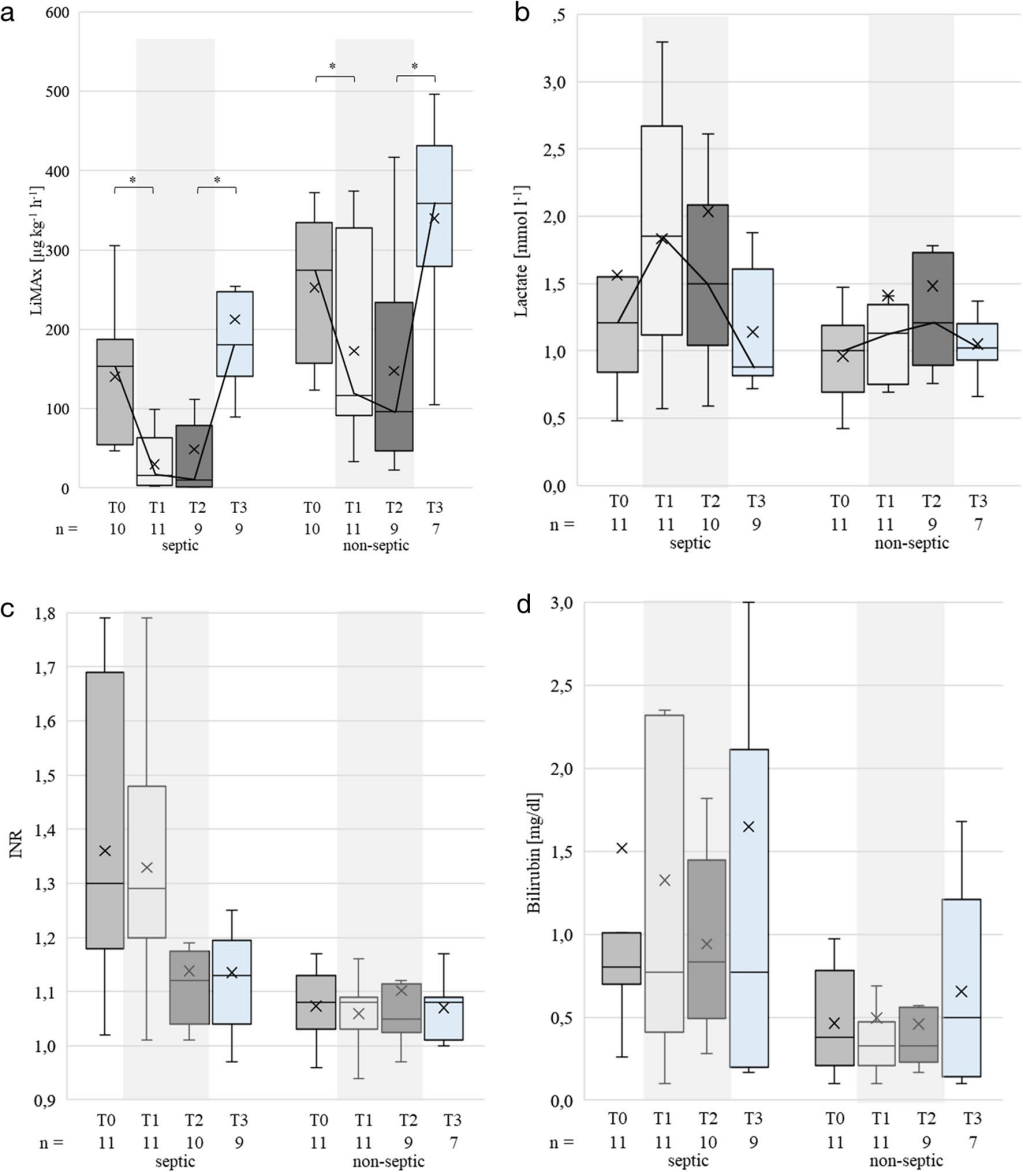
LiMAx, lactate, INR and bilirubin of septic and non-septic patients over time (T0–T3). Grey shaded area: presence of isoflurane; box-plot using the exclusive median; x: mean; T0: before isoflurane sedation; T1, T2: with isoflurane sedation as part of routine clinical care; T3: after termination of isoflurane. **a** LiMAx values. Bold line indicates mean course. **b** Lactate. **c** INR. **d** Bilirubin. *:significant differences. LiMAx: maximum liver function capacity; INR: international normalized ratio

The time courses of the LiMAx values demonstrated a pattern of both decrease and increase in behavior (*p* < 0.0001) and were similar between groups (*p* = 0.416), although in general LiMAx values were lower in sepsis patients as compared to those without sepsis (*p* < 0.001, Fig. 1a).

The mixed model analysis showed that the effects of time (T0–T3) and sepsis each were significant (*p* < 0.001). Thus both, the presence of sepsis and the presence of isoflurane, have an independent yet additive effect on reducing the LiMAx values.

The course of the lactate levels was equal between the groups (Fig. 1b) and exhibited a behavior opposite to the LiMAx values. Lactate increased with the advent of isoflurane (T1 and T2) and decreased after cessation thereof (*p* = 0.022), but lactate levels were not different between septic and non-septic patients (*p* = 0.1805). The mean of lactate levels obtained from the routine blood gas analyses for all patients increase with isoflurane from 0 to 48 h compared to the 24 h before isoflurane start (Additional file 1: Fig. S2).

## Discussion

In the present study, we found that both isoflurane and sepsis were factors that independently decreased the CYP1A2-mediated methacetin metabolization as determined by the LiMAx test system. This study was triggered by an earlier observation in three patients, who had significantly reduced LiMAx test values in the absence of any clinical significant liver dysfunction [8]. These patients stipulated our scientific interest, served as pilot patients for ethics approval and were included in this analysis.

### LiMAx values in sepsis

The mRNA expression of several drug-metabolizing CYP450 enzymes, including CYP1A2, is decreased by the pro-inflammatory cytokines IL-6, IL-1β, TNF-α and IFN-ɤ in vitro [18, 19]. The resulting cytokine-mediated decrease in CYP1A2 enzyme activity during sepsis was measured in vivo by two research groups. LiMAx values as low as 109 µg kg^−1^ h^−1^ were reported for septic patients and 65 µg kg^−1^ h^−1^ for septic shock patients early in the course of disease within 24 h after diagnosis [20]. Another study in 28 septic patients reported the lowest mean LiMAx values at 165 ± 93 µg kg^−1^ h^−1^, 2 days after sepsis onset [6].

Before and after application of isoflurane at T0 and T3, our data were similar with these findings. But during isoflurane inhalation the LiMAx values were significantly decreased to 30 ± 34 µg kg^−1^ h^−1^ (21%) and 49 ± 76 µg kg^−1^ h^−1^ (35%), respectively, in the presence of sepsis (Fig. 1a). This decrease was much more pronounced than expected and suggests that isoflurane has independently a decreasing effect on LiMAx values.

Isoflurane appears to substantially reduce the clinical validity of LiMAx values with respect to global liver function. The LiMAx test in general is a valuable tool for the assessment of global liver function. CYP1A2 represents 5–20% of the hepatic P450 pool [21]. The activity of this enzyme, measured by LiMAx testing, has been shown to correlate with global liver function [22] and the model of end stage liver disease (MELD) score, and may serve as a predictor of death in liver failure [23, 24].

In contrast, this correlation was in part removed during inhalation of isoflurane at T1 and T2 for the septic patients. The median MELD score, also including INR and bilirubin (Fig. 1c, d), at the time of diagnosis of sepsis decreased continuously indicating constant liver recovery. Thus, the clinical significance of the LiMAx test for determining global liver function in the presence of isoflurane appears to be substantially limited.

Furthermore, in the septic patient group the 28-day mortality rate of 9% does not correlate to the predicted mortality derived from the measured LiMAx values, which has been reported to be 55% for septic patients with values < 100 µg kg^−1^ h^−1^ [5]. We suggest that LiMAx test results measured during inhaled sedation with isoflurane should not be used for clinical decision-making or prediction of mortality.

### Isoflurane does not interfere with the LiMAx measurement technique

Previous LiMAx studies have excluded isoflurane treated patients because of suspected interference [2]. One hypothesis was that isoflurane could disturb physical measurement in the LiMAx device. However, no contraindications are given in the user’s manual for the “FLIP 2.0”device [25] except allergic reactions on methacetin or paracetamol. Further, in vitro addition of 3 vol% isoflurane to the LiMAx sample chamber filled with 3% CO_2_ did not alter the measured LiMAx values (Humedics GmbH, personal communication). To exclude any effect of oxygen supply on ^13^CO_2_:^12^CO_2_ ratio measurement by nondispersive isotope selective infrared spectroscopy [26] the fraction of inspired oxygen (FiO_2_) was adjusted on the basis of arterial blood gas analyses and subsequently kept constant during LiMAx measurements. We conclude that the LiMAx test results correctly indicate CYP1A2 enzyme activity also in the presence of isoflurane.

### Isoflurane may lead to lactate accumulation

The accumulation of lactate is a well-known effect of tissue hypoxia caused by the disturbance of microcirculation during sepsis [27] and has been used to define the severity of the disease, i.e., septic shock as a lactate level > 2 mmol/l [13]. In contrast, lactate levels of liver healthy patients are relatively stable. Slight fluctuations in lactate levels are buffered by resynthesis of lactate to glucose. While high lactate levels could be explained as expected from septic patients there was also an increase in lactate levels during isoflurane sedation in the non-septic patients (Fig. 1b). This effect was equally observed in both groups suggesting that there is an additive effect of isoflurane and sepsis on lactate levels likewise on CYP1A2-activity. When considering the time from 24 h before to 48 h after start of sedation an increase of lactate levels over time can be observed from the beginning of isoflurane sedation on (Additional file 1: Fig. S2).

### Network model for isoflurane-dependent decrease of CYP1A2 activity

Isoflurane has an endogenous impact on metabolism and cellular function. Exposure leads to a changed gene expression profile in liver (15% of 4900 genes), heart (approx. 3%) and kidney (approx. 4%) in mammalian (rats) using rat oligonucleotide gene arrays [28]. The volatile anesthetic mimics biologic ischemic preconditioning which was first studied 1986 by Murry et al. in an animal model [29]. It reduces myocardial necrosis after coronary artery occlusion and protects kidney, brain and liver from ischemic injury [30]. This anesthetic induced preconditioning is presumably initiated by the stabilization of hypoxia inducible factor (HIF-1α) in the presence of isoflurane [31]. Therefore, we suppose the HIF-1α to be the possible link connecting isoflurane, lactate accumulation and CYP1A2 liver enzyme activity.

**Fig. 2:**
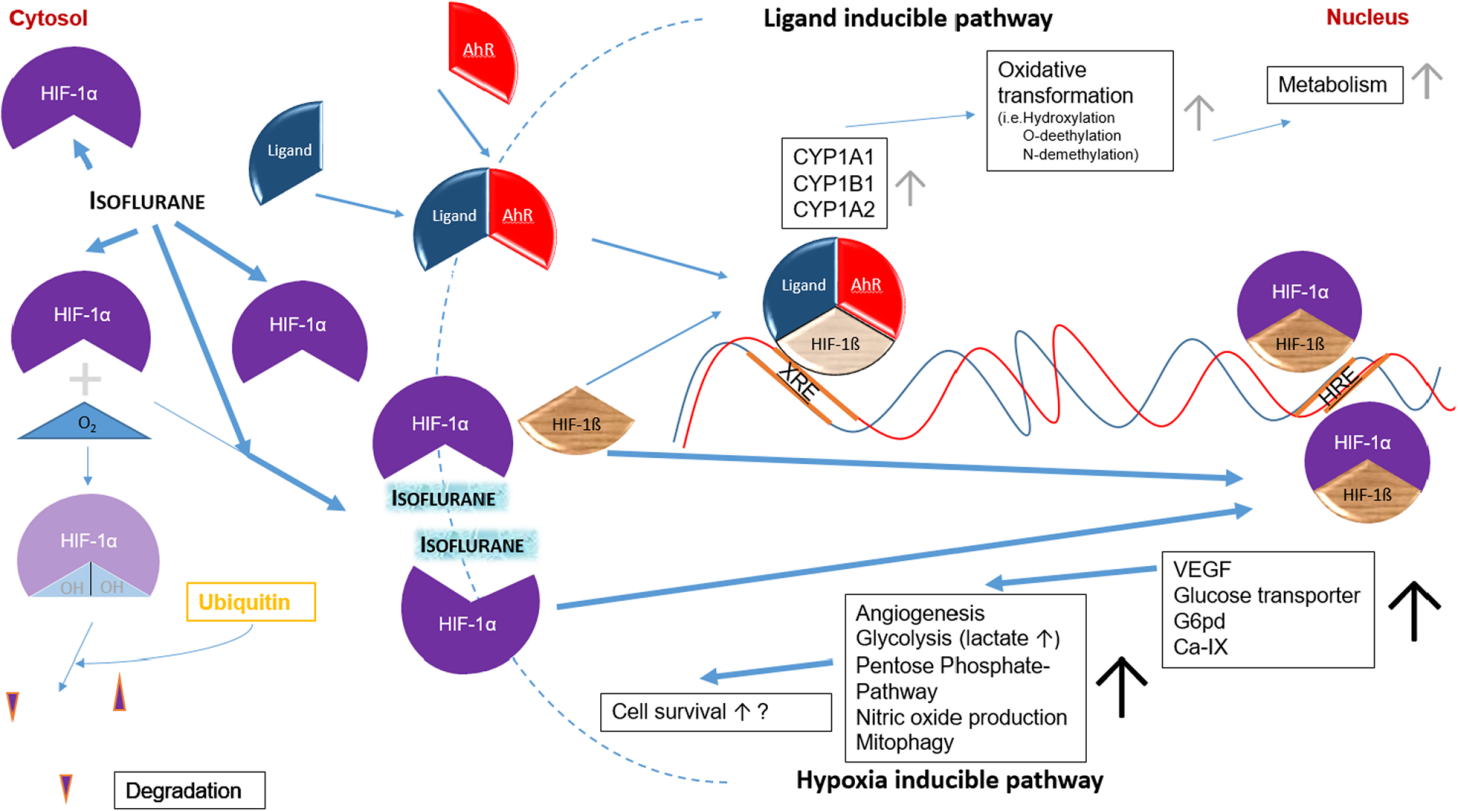
Hypothetical network model of isoflurane on oxygen dependent regulation via HIF-1α. Under normoxic conditions, HIF-1α is hydroxylated in the cytosol and degraded in an ubiquitin-dependent manner. Upon hypoxia, less HIF-1α is hydroxylated and degraded. The unhydroxylated HIF-1α translocates to the nucleus and binds to HIF-1ß to form the active transcription factor HIF-1. This binds to hypoxia responsive elements (HRE) and activate the hypoxia-inducible pathway. A reduced availability of HIF-1ß leads to downregulation of the ligand-inducible pathway. This is activated, when a ligand (e.g., polycyclic aromatic hydrocarbons, dioxins or ß-naphthoflavones) binds to the aryl hydrocarbon receptor (AhR), allowing translocation of the complex to the nucleus where it binds to HIF-1ß. The emerged transcription factor binds to xenobiotic responsive elements (XRE) in the promotor region of xenobiotic-degrading enzymes including CYP1A1, CYP1B1 and CYP1A2 enhancing their expression. Therefore, increased binding of HIF-1ß to HIF-1α under hypoxic conditions results in less active ligand/AhR/HIF-1ß transcription factor and a subsequent decrease of CYP1A1, CYP1B1 and CYP1A2 expression. Isoflurane leads to upregulation of HIF-1α protein expression, increased HIF-1α nuclear translocation [35] and DNA binding activity [36]. Isoflurane may cause a reduction of the CYP1A2 enzyme activity, measured by LiMAx, via upregulation of the hypoxia-inducible pathway and subsequent downregulation of the ligand-inducible pathway. VEGF: vascular endothelial growth factor; G6pd: glucose-6-phosphate dehydrogenase; CA-IX: carbonic anhydrase 9

Physiologically, the stability of HIF-1α is regulated via O_2_-dependent prolyl hydroxylation, which targets HIF-1α for ubiquitylation and subsequent degradation by the proteasome in aerobic conditions [32]. Under hypoxic and pro-inflammatory conditions, HIF-1α is stabilized. It plays a central role in the cellular response to low oxygen levels [32] and switches the immune metabolisms towards pro-inflammatory glycolysis [33] and forms together with HIF-1β the heterodimeric transcription factor HIF-1 that induces the expression of genes for glycolysis, angiogenesis and erythropoiesis. Isoflurane has been shown to induce time-dependent upregulation of HIF-1α expression [34] in the absence of ischemia and reperfusion. Under isoflurane more HIF-1α protein can be detected that is translocated into the nucleus in mammalian heart tissue [35, 36]. Subsequently, the DNA binding activity of the transcription factor HIF-1 is increased [36]. Upon HIF-1α stabilization, HIF-1 binds to hypoxia-response elements (HREs) to induce the transcription of proteins involved in erythropoiesis, vascularization, glucose uptake and glycolysis including lactate dehydrogenase-A [37]. While oxidative decarboxylation of pyruvate leads to acetyl-CoA under aerobic conditions, lactate dehydrogenase dehydrates pyruvate to lactate as part of the anaerobic glucose-metabolism [38]. The reactions towards acetyl-CoA is hampered by HIF-1α and in turn the generation of lactate is enhanced by activating lactate dehydrogenase A [33]. In vivo, CYP1A2 enzyme activity underlies strong environmental modulations, i.e., oxygen availability [39]. It is regulated via HIF-1α stabilization that affects the aryl hydrocarbon receptor (AhR) network and leads to the metabolism of xenobiotics [40]. The pathway is activated by a heterodimer of the ligand-activated transcription factor AhR and HIF-1β. Because HIF-1α and AhR compete for HIF-1β, HIF-1α activation attenuates AhR-mediated gene expression. CYP1A2 expression depends on the HIF-1α-dependent formation of the heterodimeric transcription factor AhR/HIF-1β (Fig. 2). The bidirectional promotor region of *CYP1A1* and *CYP1A2* contains at least 13 *AHR* response elements [41]. As shown in a cell culture sepsis model, downregulation of AhR results in a decreased CYP1A2 expression in hepatocytes [42].

We combined the results of the in vitro and in vivo studies available in the literature with our findings and developed a hypothetical network model. We suppose that isoflurane sedation in humans decreases LiMAx values because it stabilizes the transcription factor HIF-1α. The downstream effects of this stabilization include the reduced CYP1A2-activity and increased lactate production (Fig. 2). We acknowledge the pure hypothetical status of this model, which needs to be investigated further in basic science.

### Clinical implications

This study was stipulated by the clinical observation that LiMAx values were considerably lower during isoflurane sedation that was not coherent with the liver function assessed otherwise. It provides a theory linking the findings of bench (cell culture and animal model) studies and clinical data obtained during treatment of intensive care patients. Downregulation of CYP1A2 and possibly other cytochromes by isoflurane may change, i.e., slow down the kinetics of drug metabolism significantly. This has to be taken into account when selecting drugs used for the treatment, drug dosing and the evaluation of treatment effect or unwanted side effects. Since the LiMAx test does not correlate well with global liver function during isoflurane administration, we suggest that the test should not be used to draw clinical conclusions if inhaled sedation is used.

### Limitations

Since there is no gold standard or reference method for the assessment of liver function the MELD score is often used to characterize the status of liver function. It was used by us in exact this regard to characterize liver function by conventional lab testing (INR and bilirubin are included) but not to make predictions. The aim of this study was to investigate the influence of isoflurane on functional liver testing by LiMAx and provide a theoretical explanation for the observed effects. The sample size of 22 patients, although suitable for an observational study, may limit the generalizability of the findings while the retrospective data could introduce selection bias and challenges in matching and analyzing the data. The absence of a control group limits the ability to assess the specific impact of isoflurane on LiMAx test results. The hypothesis generated is based on the presented study data and the current knowledge available in the literature from a variety of molecular studies. Further studies would be warranted to confirm the effect of isoflurane on decreasing the LiMAx measurements. This study proposes a potential mechanism involving HIF-1α stabilization and downregulation of the ligand-inducible pathway.

## Conclusions

The presented data show that sepsis and isoflurane have independent and additive effects on reducing the hepatic CYP1A2-activity. We propose that the hypoxia-induced upregulation of the hypoxia-inducible pathway and the downregulation of CYP1A2-activity via the ligand-inducible pathway is increased by the HIF-1α-stabilizing effect of isoflurane, mimicking hypoxia. The accumulation of lactate during isoflurane sedation in patients without septic hypoxia indicates anaerobic metabolism and supports this assumption. Whether the reduced CYP1A2-activity, measured by LiMAx testing, is an adequate correlate to global liver function in patients with isoflurane sedation, should be validated further. Until then, clinical implications regarding global liver function and mortality should not be taken on the basis of LiMAx test results.

### Supplementary Information

**Additional file 1: Fig. S1.** Numbers of individuals at each stage of study inclusion process. **Fig. S2.** Lactate over time. Lactate values were available every 2-6 h from routine blood gas analysis. Each depicted time point includes lactate data of a 4-h time window (± 2 h). The data of 1 septic patient and 1 non-septic patient who died during sedation with isoflurane were omitted.

## Data Availability

The datasets analyzed during the current study are available from the corresponding author on reasonable request.
