# Supplementary material for: Isoflurane, like sepsis, decreases CYP1A2 liver enzyme activity in intensive care patients: a clinical study and network model
Source: Intensive Care Med Exp. 2024 Apr 8;12:33. doi: 10.1186/s40635-024-00617-8 (PMC11001842; doi:10.1186/s40635-024-00617-8)
Supplement: Supplementary file 1 — Additional file 1: Fig. S1. Numbers of individuals at each stage of study inclusion process. Fig. S2. Lactate over time. Lactate values were available every 2-6 h from routine blood gas analysis. Each depicted time point includes lactate data of a 4-h time window (± 2 h). The data of 1 septic patient and 1 non-septic patient who died during sedation with isoflurane were omitted. [file 40635_2024_617_MOESM1_ESM.docx]

Mechanically ventilated patients between 04/2019 and 01/2021

N = 687

Patients with only intravenous sedation

N = 522

Patients with planned or implemented sedation-change to an inhaled regime

N = 165

Patients without sedation-change

N = 78

Patients with sedation-change to an inhaled regime (planned or implemented)

N = 87

Patients without clinically indicated LiMAx measurement

N = 64

Patients with sedation-change to an inhaled regime and clinically indicated LiMAx measurement

N = 23

Patients with severe preexisting liver damage

N = 1

Patients with sedation-change and installed LiMAx measurement

N = 22

**Fig. S1.** Numbers of individuals at each stage of study inclusion process

 Fig. S2. Lactate over time. Lactate values were available every 2-6h from routine blood gas analysis. Each depicted time point includes lactate data of a 4 h time window (±2h). The data of 1 septic patient and 1 non-septic patient who died during sedation with isoflurane were omitted.
